# Tocilizumab as a treatment tool for ROSAH syndrome: a case report

**DOI:** 10.1186/s12348-025-00550-1

**Published:** 2025-11-21

**Authors:** Rita Teixeira-Martins, Ana Faria-Pereira, Ana Sofia Figueiredo, Renata d’Oliveira, Iva Brito, Mariana Jorge Rodrigues, Luís Figueira

**Affiliations:** 1 Department of Ophthalmology, Unidade Local de Saúde de São João, Porto, Portugal; 2 Department of Pediatrics, Unidade Local de Saúde de Trás-os-Montes e Alto-Douro, Vila Real, Portugal; 3 Service of Medical Genetics, Unidade Local de Saúde de São João, Porto, Portugal; 4 Pediatric and Young Adult Rheumatology Unit, Unidade Local de Saúde de São João, Porto, Portugal; 5 Faculty of Medicine, University of Porto, Porto, Portugal; 6 Unit of Pharmacology and Therapeutics, Department of Biomedicine, Faculty of Medicine, University of Porto, Porto, Portugal; 7 MedInUP - Center for Drug Discovery and Innovative Medicines, Faculty of Medicine, University of Porto, Porto, Portugal; 8 Department of Ophthalmology of Local Health Unit of São João, Avenida Prof. Hernâni Monteiro, Porto, 4202–451 Portugal

**Keywords:** ROSAH syndrome, Tocilizumab, Immunosuppression, Children

## Abstract

**Background:**

ROSAH syndrome (Retinal dystrophy, Optic nerve edema, Splenomegaly, Anhidrosis, and Headache) is a rare autosomal dominant disorder caused by heterozygous missense pathogenic variants in *ALPK1*. This gene encodes alpha-kinase 1, a key regulator of the inflammatory NF-κB pathway. Pathogenic variants in *ALPK1* are associated with increased NF-κB activation and chronic inflammation. Clinically, ROSAH syndrome presents with early-onset retinal dystrophy and optic nerve edema, alongside systemic inflammatory and non-inflammatory features.

**Case presentation:**

We present a 16-year-old female diagnosed with ROSAH syndrome, genetically confirmed. Her clinical course began with subtle abnormalities in her hands by 18 months of age, and by the age of 2 years-old (YO) it was suspected of acquired arthrogryposis and advanced bone age. Around the age of 4YO she was diagnosed with bilateral macular and papillary edema and despite treatment with methotrexate and adalimumab, she continued to experience persistent ocular inflammation and progressive vision loss; hands and feet deformities stopped evolving around age of 6-7YO; at 12YO, due to worsening of macular edema and optic disc swelling, her treatment regimen was modified to include rituximab, which led to modest improvements. Her diagnostic odyssey culminated at 15YO with the identification of the pathogenic variant p.Tyr254Cys in *ALPK1*. Recently, tocilizumab, an IL-6 receptor antagonist, was added, resulting in significant reduction in macular edema and optic disc swelling just within one month. However, visual function did not improve due to pre-existing retinal damage.

**Conclusion:**

Tocilizumab showed significant anatomical benefit in this ROSAH syndrome patient, suggesting a role for IL-6 in disease pathophysiology. Although vision could not be restored, the response supports the use of targeted biologics in managing ROSAH. Broader clinical studies are needed to confirm efficacy. This case also highlights the importance of including *ALPK1* in genetic panels for optic neuropathies, retinal disorders, and unexplained arthropathies to improve diagnosis and treatment strategies.

## Background

ROSAH (Retinal dystrophy, optic nerve edema, splenomegaly, anhidrosis, and headache) syndrome is a rare autosomal dominant autoinflammatory disorder characterized by a constellation of manifestations, including the ones that compose its name acronym [1, 2]. To date, only three heterozygous missense pathogenic variants in the *ALPK1* gene, have been described to cause ROSAH [the recurrent variant c.710 C >T (p.Thr237Met), c.761 A >G (p.Tyr254Cys) and the more recently published c.830 C >T (p.Ser277Phe)], *ALPK1* gene encodes the alpha-kinase 1 protein [3–5] which is a crucial component of the innate immune system, where it functions as a receptor for bacterial sugars, such as ADP-heptose, and activates the inflammatory NF-κB signaling pathway. The known pathogenic variants in *ALPK1*, particularly those that lead to a gain of function, result in persistent activation of the NF-κB pathway, contributing to the broad spectrum of inflammatory symptoms seen in ROSAH syndrome [1, 6, 7].

The clinical presentation of ROSAH syndrome is diverse, with patients typically exhibiting significant ophthalmic abnormalities, such as early-onset retinal dystrophy and optic nerve edema, which can lead to progressive vision loss. The ophthalmic features of the condition include early-onset optic disc edema with progressive vision loss, particularly affecting central vision and color perception, often in the context of cone or cone-rod dystrophy (CORD). Patients may exhibit varying levels of intraocular inflammation in the form of anterior chamber or vitreous cell on examination. Common symptoms include decreased vision due to optic nerve edema, anterior and posterior uveitis and a cone-rod pattern of visual impairment, which can become severe by the third decade of life. Additional findings include vitreous hemorrhage, intraretinal hemorrhage, macular edema, retinal neovascularization, cystic retinal changes, retinoschisis, and retinal degeneration. OCT imaging often shows disruptions in the retinal pigment epithelial layer and the ellipsoid zone, while full-field electroretinography (ffERG) tests reveal reduced scotopic responses [1, 3, 4, 8].

Additionally, being an autoinflammatory disease, the syndrome is associated with systemic inflammatory symptoms, including recurrent fevers, fatigue, deforming arthritis, cyclical cytopenias, meningeal and/or central nervous system inflammation and elevated inflammatory markers. However, ROSAH syndrome also includes features not typically linked to inflammation, such as dental and nail abnormalities, including hypoplasia of dental enamel, short dental roots, and inability to sweat or lactate, suggesting a more complex underlying pathophysiology [4].

Despite its rarity, there is growing interest in the potential for targeted immunomodulatory therapies to manage ROSAH syndrome. Tocilizumab, an interleukin-6 (IL-6) receptor antagonist, has shown promise in treating other autoinflammatory disorders by mitigating the effects of chronic inflammation. However, its efficacy in ROSAH syndrome remains underexplored [4].

In this case report, we present the clinical course of a 16 YO female patient with ROSAH syndrome, after a diagnostic odyssey of more than 10 years and which first clinical signs were hands arthropathy, who recently has been under tocilizumab treatment. The patient exhibited significant improvement in ophthalmic symptoms, highlighting the potential of tocilizumab as an effective treatment option for this challenging condition. This clinical case description adds to the growing body of evidence supporting the role of targeted therapies in managing ROSAH syndrome, offers insights into the broader implications of IL-6 blockade in autoinflammatory diseases, and helps expanding ROSAH phenotype.

## Case presentation

A 16-year-old female patient is presented, with a complex medical history characterized by inflammatory ocular and musculoskeletal clinical manifestations, leading to a recent diagnosis of ROSAH syndrome (Retinal dystrophy, Optic nerve edema, Splenomegaly, Anhidrosis, and Headaches) confirmed by the identification of the pathogenic variant c.761 A > G (p.(Tyr254Cys)) in *ALPK1* (NM_025144.4). The patient’s clinical journey began with a normal, uneventful pregnancy and birth at 38 weeks of gestation. The neonatal period was uncomplicated, attaining appropriate growth and psychomotor development milestones in early childhood.

At 18 months of age, subtle abnormalities in hand function were noted by her caregivers. By the age of 2 years, she developed signs suggestive of acquired arthrogryposis, and metaphyseal chondrodysplasia was suspected (due to contractures of the small joints of the fingers, which later involved the wrists, knees, and feet). Initial manifestations included difficulty in full extension, followed by painless swelling of the proximal interphalangeal joints and knees, without significant morning stiffness. During this time, inflammatory markers and autoantibodies consistently returned negative results.

At the age of 4 years, she started with vision problems (see full description ahead).

At the age of 8 years, she presented normal height (50th percentile) and painless swelling of all interphalangeal joints with palpable nodules, restricted finger extension and camptodactyly, as well as dystrophic nails. Additionally, there was bilateral limitation of elbow extension, bilateral restricted knee extension, and bilateral cavum feet with global ankylosis, along with deformities of the toes.

### Diagnostic evaluation

The patient underwent an extensive diagnostic workup, including genomic chromosomal analysis, single-gene analysis, and trio whole-exome sequencing (WES), which found no pathogenic variants. Echocardiogram, renal and abdominal ultrasounds, and magnetic resonance imaging (MRI) of the brain showed no abnormalities. Further investigations, including electromyography (EMG) and cerebrospinal fluid (CSF) analysis, were also normal. A metabolic screen for lysosomal storage disorders returned negative results.

Radiographs revealed metaphyseal changes suggestive of a possible bone dysplasia, worsening progressively. Despite being treated with methotrexate for suspected inflammatory arthritis, the patient did not show a significant therapeutic response.

More recently, after ROSAH syndrome was suspected, *ALPK1* analysis by Next Generation Sequencing (NGS) (re-analysis of exome sequence capture), and the pathogenic variant c.761 A > G (p.(Tyr254Cys)) was identified, confirmed to be *de novo*, after targeted parental analysis. This genetic confirmation was obtained when the patient was 15 years old, approximately one year before initiating tocilizumab therapy.

### Surgical interventions

**Fig. 1:**
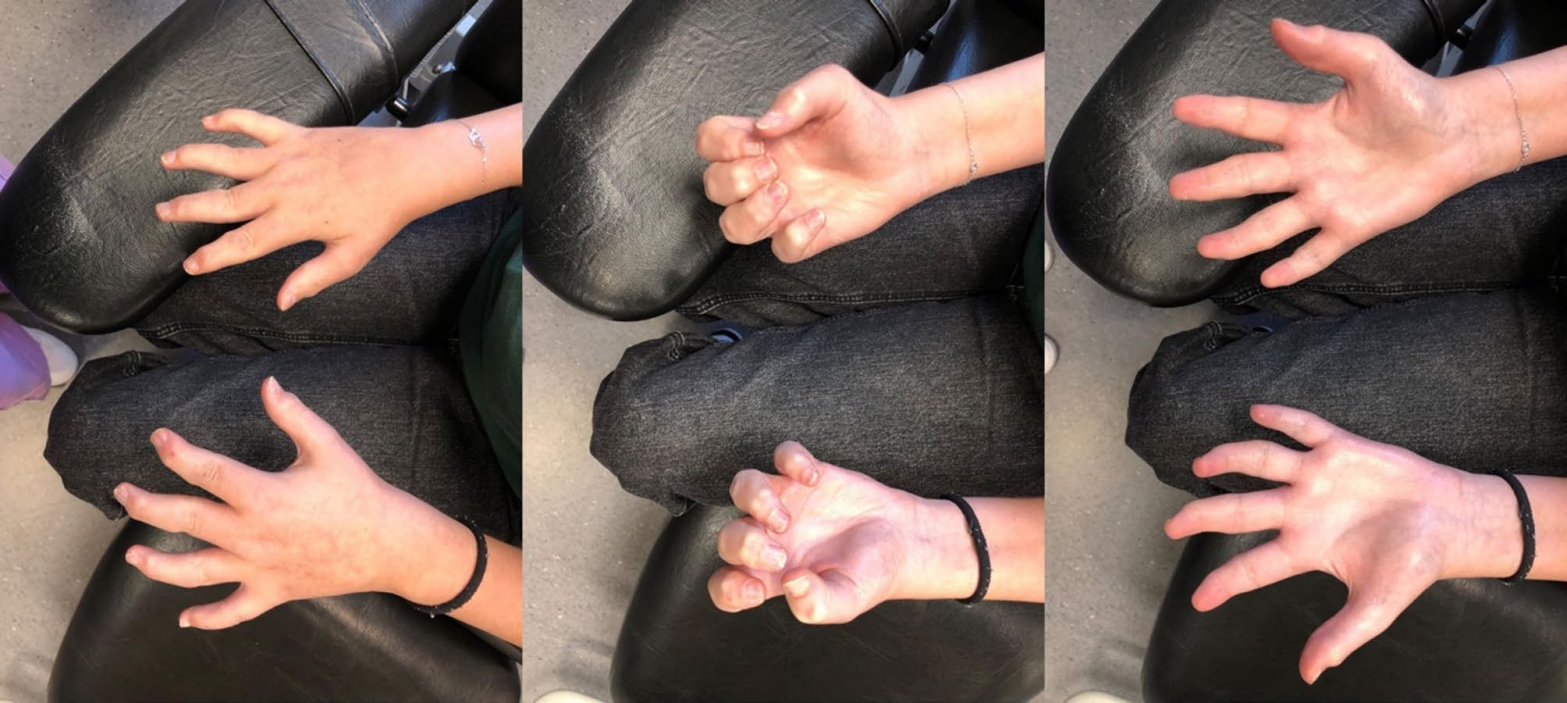
Inflammatory arthritis involving wrist, metacarpophalangeal and interphalangeal joints with evolving joint deformities of both hands

Orthopedic interventions over the years included V tarsectomy and calcaneal osteotomy at the age of 9 years, knee hemi-epiphysiodesis with 8-plates in at the age of 12 years and triple arthrodesis of the left and right foot in the age of 13 and 14 years, respectively. Despite functional improvement, the patient continues to exhibit nail dystrophy and limitations in joint mobility, particularly in the elbows, wrists, fingers, and feet, with notable deformities including camptodactyly (Fig. 1) in fingers and toes, with *cavus* feet.

### Ophthalmologic findings

**Fig. 2:**
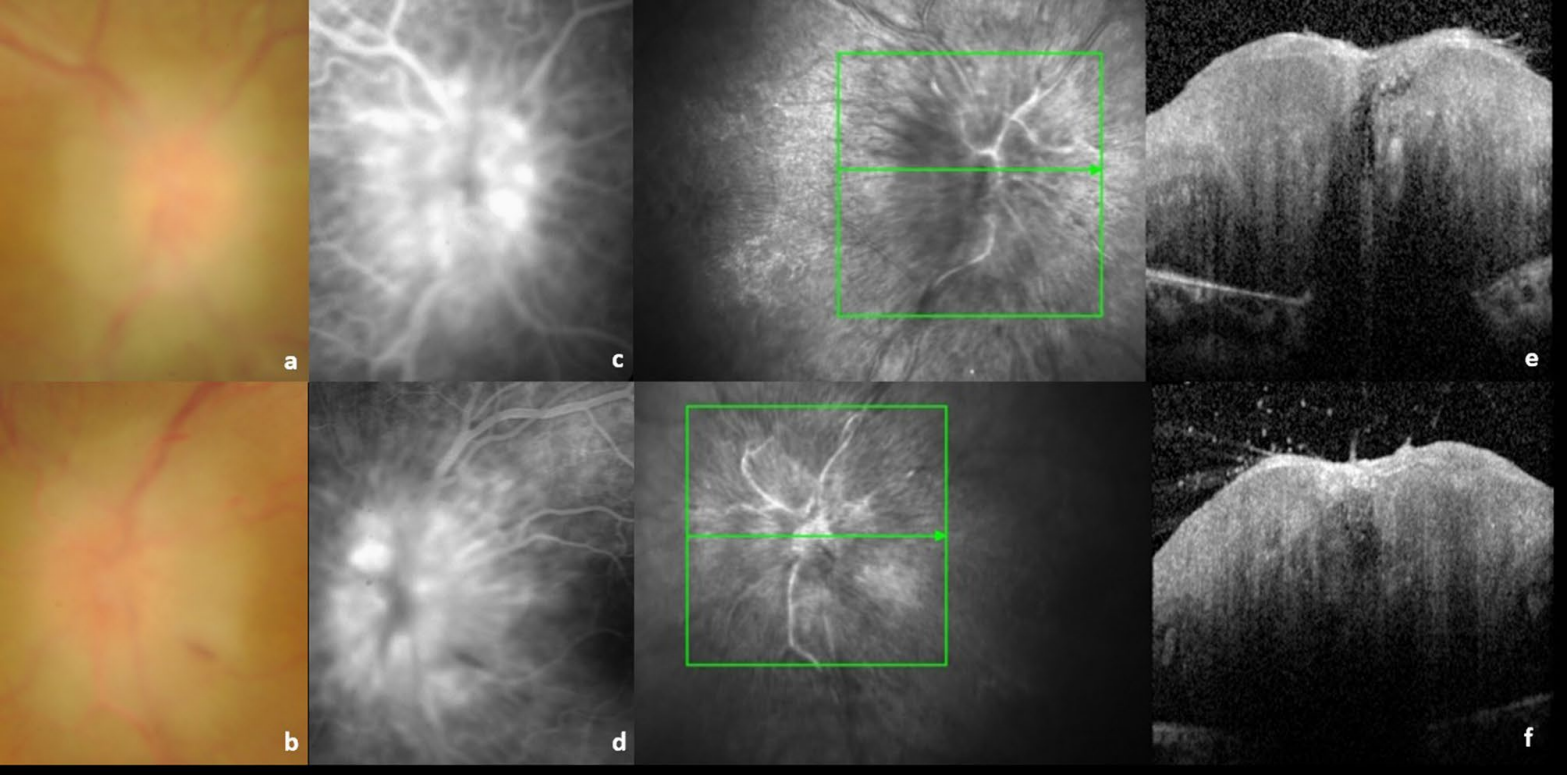
OD (**a**) and OS (**b**) colour fundus photographs revealing hyperemic and elevated optic discs with associated peripapillary hemorrhages and blurred contours; OD (**c**) and OS (**d**) FA revealing papillary leakage in early stages; SD-OCT images of the right (**e**) and left (**f**) optic nerve head demonstrates a severe optic nerve edema, with marked thickening and swelling of the nerve fiber layer. Abbreviations: OD, right eye; OS, left eye; FA, fluorescein angiography; SD-OCT, Spectral-Domain Optic Coherence Tomography

Ophthalmologic abnormalities became evident at the age of 4 years when the patient presented with bilateral optic disc edema associated with macular edema, noted on fundoscopy. Best corrected visual acuity (BCVA) at the time of initial ophthalmologic examination was recorded as 0.1 logMAR in the right eye (OD) and 0.2 logMAR in the left eye (OS). Slit-lamp examination revealed a mild flare in the anterior chamber, with no other ocular abnormalities. Fundus examination revealed hyperemic and elevated optic discs with associated peripapillary hemorrhages, but without evidence of active uveitis, vasculitis, or retinitis (Fig. 2a and b).

**Fig. 3:**
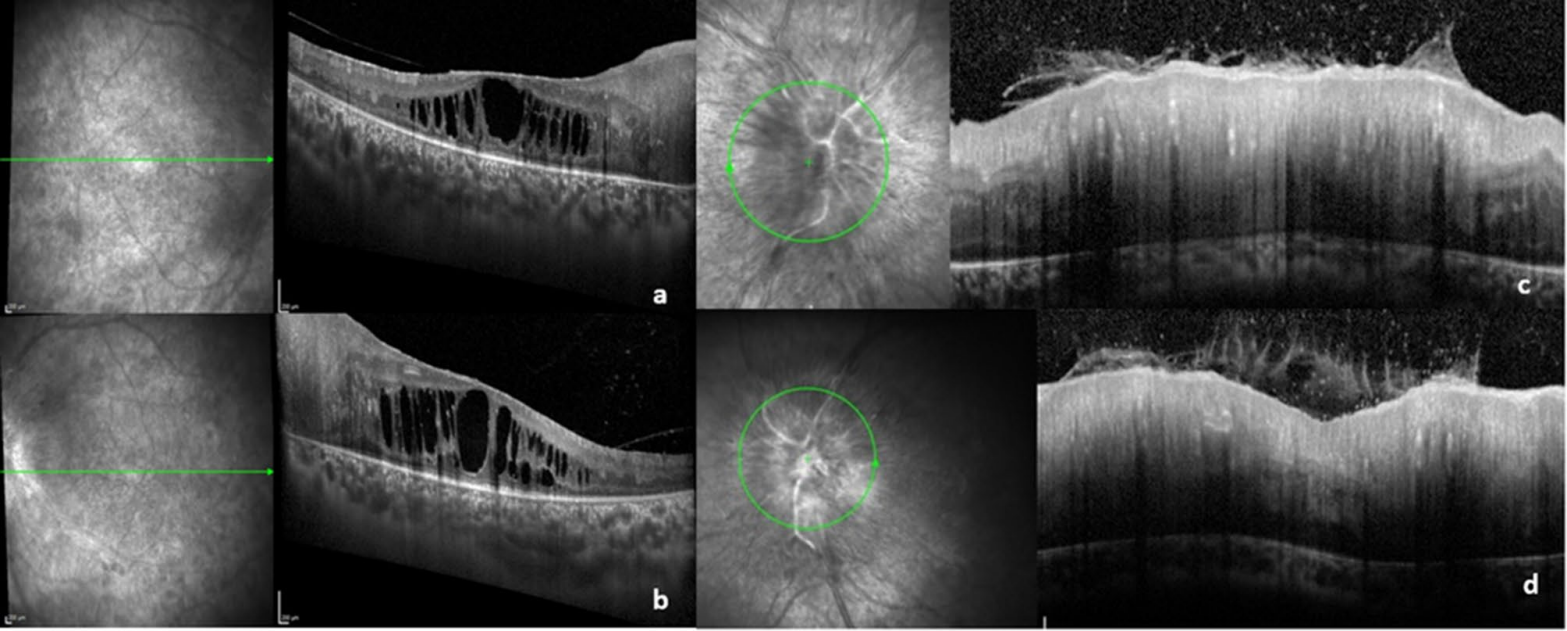
OD (**a**) and OS (**b**) macular SD-OCT reveal severe cystoid macular edema, characterized by large, fluid-filled cystic spaces within the macular region. SD-OCT images of the right (**c**) and left (**d**) optic nerve head demonstrate marked thickening and swelling of the retinal nerve fiber layer. Multiple intraretinal hyperreflective foci are visible, consistent with active inflammatory activity. Abbreviations: OD, right eye; OS, left eye; SD-OCT, Spectral-Domain Optical Coherence Tomography

As she grew older and became more capable of cooperating with supplementary diagnostic tests, it became possible to conduct serial Spectral-Domain Optic Coherence Tomographies (SD-OCT), which revealed significant thickening of the retinal nerve fiber layer (RNFL) (Figs. 2e and f and 3c and d) and the presence of cystoid macular edema (CME) in OD and OS at the age of 8 (Fig. 3a and b). Fluorescein angiography (FA) demonstrated intense and early peripapillary leakage but ruled out active retinal vasculitis or neovascularization (Fig. 2c and d). B-scan ultrasonography revealed a thickened scleral wall with a T-sign, which confirmed posterior scleritis.

The initial management involved systemic corticosteroid therapy, which provided an incomplete response. Methotrexate, and later adalimumab, were introduced due to the suspected inflammatory nature of the ocular findings. Methotrexate was administered at a dose of 10 mg/m² per week, and adalimumab was given subcutaneously at a dose of 40 mg every two weeks, in accordance with standard pediatric uveitis protocols. Despite these interventions, the macular edema persisted, and there was a continued decline in visual acuity, particularly in the OD. Adalimumab serum levels remained within the therapeutic range (9.8 µg/mL), and anti-adalimumab antibodies were negative. Over time, the patient developed a chronic, progressive form of macular edema, which, along with persistent optic disc edema, contributed to a gradual decline in visual function. Slit-lamp examination consistently revealed a mild flare in the anterior chamber throughout follow-up, but no cells were ever observed. The patient’s visual acuity deteriorated to 1.0 logMAR in OD and 0.3 logMAR in OS at the age of 12, prompting a reevaluation of her management strategy. The decision was made to switch to rituximab (RTX), an anti-CD20 monoclonal antibody, with the aim of targeting B-cell-mediated inflammation. This treatment led to a modest reduction in macular edema. However, the response was not adequate to prevent further visual decline.

At age 14, a clinical suspicion of the recently described ROSAH syndrome led to its confirmation by reviewing her previous WES. This, alongside non-responsive optic nerve and macular edema to conventional treatments, necessitated exploring novel therapeutic approaches.

**Fig. 4:**
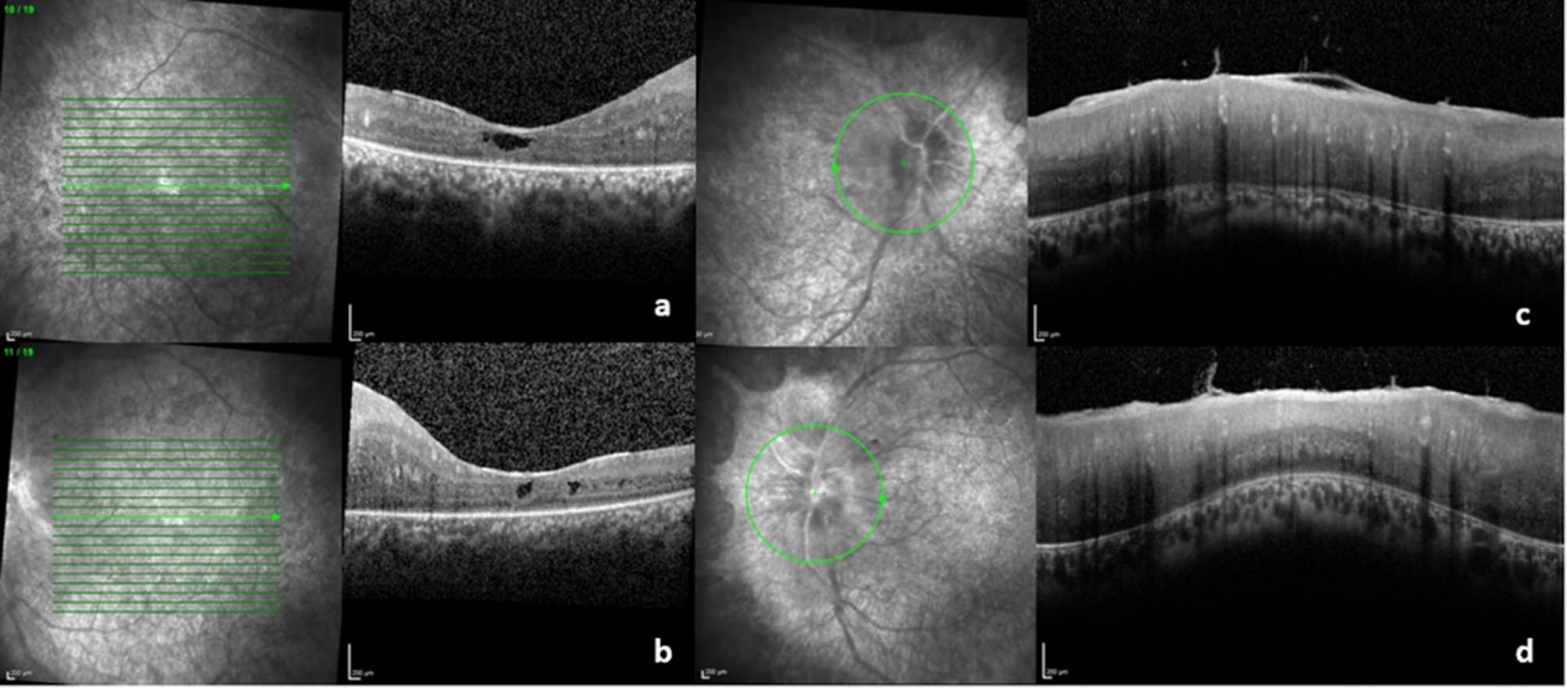
OD (**a**) and OS (**b**) macular SD-OCT show a clear reduction of cystoid macular edema as well as signs of retinal outer layer atrophy. SD-OCT images of the right (**c**) and left (**d**) optic nerve head demonstrate a reduction in optic nerve edema. The previously noted hyperreflective foci have nearly disappeared after treatment, indicating resolution of intraretinal inflammatory activity. Abbreviations: OD, right eye; OS, left eye; SD-OCT, Spectral-Domain Optic Coherence Tomography

Following previous reports of success, after multidisciplinary discussion, monthly intravenous tocilizumab was initiated at 8 mg/kg. Remarkably, one month after initiating tocilizumab therapy, the patient exhibited significant clinical improvement. OCT imaging demonstrated a substantial reduction in macular edema (Fig. 4a and b), with a significant decrease of central macular thickness compared to baseline, as well as a notable reduction in optic disc swelling (Fig. 4c and d), suggesting and effective control of the disease process. Despite clear anatomical response, there was no functional improvement due to atrophy of the retinal outer layers, consistent with the prolonged disease process. Intraocular pressure has remained stable.

This patient never exhibited recurrent fevers or elevated inflammatory markers, anhidrosis, cytopenias or splenomegaly. After starting tocilizumab, the patient reported improvement in joint stiffness and fatigue, but musculoskeletal findings remain unchanged. Treatment was started when the patient was 16 years old and has now been ongoing for approximately one year, during which she has remained clinically and anatomically stable, with no recurrence of macular or optic disc edema and no adverse effects observed.

## Discussion

The ophthalmic manifestations of ROSAH syndrome are complex, and the potential causes of vision loss are multifactorial. *Huryn et al.*. propose that three main factors contribute to visual function changes in ROSAH syndrome patients: optic nerve involvement, ocular inflammation including cystoid macular edema, and retinal degeneration [1]. 

Currently, there is no consensus on the treatment for ROSAH syndrome. According to a recent report by *Kozycki et al.*., 7 out of 10 patients treated with biologics reported subjective improvements in systemic symptoms, and 3 out of 4 patients receiving adalimumab had normalized C-reactive protein levels [4]. *Zhong et al.*. observed improvements in uveitis with adalimumab therapy, although persistent optic disc edema and progressive visual deterioration were also noted [8]. Williams et al. have described uveitis in ROSAH syndrome as largely unresponsive to immunomodulatory therapies [6]. *Huryn et al.*. proved tocilizumab has greater efficacy for uveitic cystoid macular edema compared to TNF-α inhibitors [1]. 

Although ocular involvement is usually present, there have been some adult patients described with other inflammatory features (recurrent fevers, malaise, episodic abdominal pain, headaches, transient cytopenias) and few ocular manifestations. Interestingly patients also present some other symptoms like inability to sweat, deficient saliva and breastmilk production, but the cause is still unclear [4]. 

This case illustrates the complexity of managing inflammatory ocular conditions associated with systemic diseases like ROSAH syndrome, particularly when traditional therapies prove inadequate. Tocilizumab demonstrated notable efficacy in reducing macular edema and optic disc swelling in this patient, although without accompanying visual improvement, indicating that IL-6 may play a critical role in the pathophysiology of the condition. The current follow-up period under tocilizumab is still short (< 12 months), but the early anatomical response observed within one month suggests that IL-6 blockade may effectively suppress the underlying inflammatory component of ROSAH syndrome. However, due to the advanced stage of the disease, the full potential benefit of this immunomodulatory therapy could not be determined. Tocilizumab has also been reported to improve musculoskeletal findings which could not be identified in this patient with advanced deformities, despite a short follow-up.

Administering tocilizumab for uveitis in the early stages of ROSAH syndrome could be beneficial, but further research involving a larger patient cohort is necessary to validate these findings. Moreover, the occurrence of retinal degeneration and optic disc changes independent of intraocular inflammation raises questions about the complete efficacy of tocilizumab in this context. The primary limitation of this report is its short follow-up period and the fact that it represents a single case, which restricts the generalizability of the findings. Nevertheless, the detailed clinical, genetic, and imaging characterization provides valuable insights that may guide future studies on ROSAH syndrome.

Accurate diagnosis of this rare syndrome is essential, as patients frequently present to ophthalmologists with visual symptoms. This report also underscores the importance of including *ALPK1* in multi-gene panel analysis for Optic Neuropathy and inherited retinal diseases as well as for arthropathies of unknown etiology (juvenile idiopathic arthritis-like), in order to shorten diagnostic odyssey, improve diagnostic accuracy, management and outcomes strategies.

## Conclusion

The introduction of tocilizumab resulted in a notable improvement in macular and papillary edema in a patient with ROSAH syndrome, providing a promising therapeutic avenue for managing complex ocular inflammation in similar patients. This case adds to the growing evidence supporting the role of biologic agents targeting specific inflammatory pathways in managing refractory ocular inflammatory diseases.

## Data Availability

No datasets were generated or analysed during the current study.

## Abbreviations

ADP: Adenosine diphosphate
ALPK1: Alpha kinase 1
BCVA: Best corrected visual acuity
CME: Cystoid macular edema
CORD: Cone or cone–rod dystrophy
CSF: Cerebrospinal fluid
EMG: Electromyography
FA: Fluorescein angiography
ffERG: Full–field electroretinography
IL-6: Interleukin–6
logMAR: Logarithm of the minimum angle of resolution
MRI: Magnetic resonance imaging
NGS: Next generation sequencing
NF-κB: –Nuclear factor kappa–light–chain–enhancer of activated B cells
OCT: Optical coherence tomography
OD: Oculus dexter (right eye)
OS: Oculus sinister (left eye)
RNFL: Retinal nerve fiber layer
ROSAH: Retinal dystrophy, optic nerve edema, splenomegaly, anhidrosis, and headache
RTX: Rituximab
SD-OCT: Spectral–domain optical coherence tomography
WES: Whole exome sequencing
